# Multiple specialised goose-type lysozymes potentially compensate for an exceptional lack of chicken-type lysozymes in Atlantic cod

**DOI:** 10.1038/srep28318

**Published:** 2016-06-21

**Authors:** Marit Seppola, Kathrine Ryvold Bakkemo, Helene Mikkelsen, Bjørnar Myrnes, Ronny Helland, David M. Irwin, Inge W. Nilsen

**Affiliations:** 1 Department of Medical Biology, UiT-The Arctic University of Norway, Tromsø, Norway; 2 Pharmaq AS, Tromsø, Norway; 3 The Northern Norway Regional Health Authority, Tromsø, Norway; 4 Nofima, Tromsø, Norway; 5 Department of Chemistry, UiT-The Arctic University of Norway, Tromsø, Norway; 6 Laboratory Medicine & Pathobiology, University of Toronto, Toronto, Ontario, Canada; 7 Faculty of Health Sciences, UiT-The Arctic University of Norway, Tromsø, Norway

## Abstract

Previous analyses of the Atlantic cod genome showed unique combinations of lacking and expanded number of genes for the immune system. The present study examined lysozyme activity, lysozyme gene distribution and expression in cod. Enzymatic assays employing specific bacterial lysozyme inhibitors provided evidence for presence of g-type, but unexpectedly not for c-type lysozyme activity. Database homology searches failed to identify any c-type lysozyme gene in the cod genome or in expressed sequence tags from cod. In contrast, we identified four g-type lysozyme genes (LygF1a-d) constitutively expressed, although differentially, in all cod organs examined. The active site glutamate residue is replaced by alanine in LygF1a, thus making it enzymatic inactive, while LygF1d was found in two active site variants carrying alanine or glutamate, respectively. *In vitro* and *in vivo* infection by the intracellular bacterium *Francisella noatunensis* gave a significantly reduced LygF1a and b expression but increased expression of the LygF1c and d genes as did also the interferon gamma (IFNγ) cytokine. These results demonstrate a lack of c-type lysozyme that is unprecedented among vertebrates. Our results further indicate that serial gene duplications have produced multiple differentially regulated cod g-type lysozymes with specialised functions potentially compensating for the lack of c-type lysozymes.

The vertebrate immune system has evolved two defense systems in response to infectious agents; the innate and the adaptive immune response. Innate immunity encompasses a large set of first-line defense factors also including antimicrobial peptides and enzymes. The adaptive system involves the on-set of serial responses resulting in a long-term memorised defence including production of specific antibodies and T-cells (reviewed in^1^).

Several studies have revealed that the Atlantic cod, *Gadus morhua*, possess a unique immune system. Two decades ago, it was shown that the immunoglobulin concentration in cod serum is higher than in fish such as Atlantic salmon (*Salmo salar*)^2^. In 2005, it was hypothesised that the histocompatibility complex class II (MHC_II_) was absent^3^, which was later confirmed by genome sequencing^4^. MHC_II_ is an acknowledged key element in the humoral immune response and in vaccine efficacy, yet cod, despite the loss of MHC_II_, produce specific antibodies including a strong antibody response to intracellular bacteria like *Francisella noatunensis*^5^. In parallel to the finding of a missing MHC_II_, a highly expanded family of histocompatibility complex class I (MHC_I_) genes was identified^4^. Additionally, cod is the first sequenced vertebrate identified to have lost all orthologs of the mammalian cell surface bacterial recognising Toll-like receptor (TLR) genes and instead harbour an expanded ‘fish-specific’ TLR family^6^,^7^ that could, at least partly, compensate for the abnormalities in the innate immune system. In spite of these genetic irregularities, cod is not especially susceptible to common fish diseases although francisellosis, a granulomatous disease caused by the facultative intracellular bacterium *F. noatunensis,* is an emerging disease of particular relevance to the aquaculture sector^8^.

For decades, the designation lysozyme was typically perceived as a synonym for the chicken (c-) type, which was originally found in the hen egg white. Although the goose (g-) type lysozyme was discovered more than 50 years ago^9^, g-types were believed to be exclusive to avian species. In 2001, it was revealed that g-type lysozymes also exist in fish^10^, the first such finding in non-bird species. Indications of a wider distribution of g-type lysozymes came by the finding of g-type genes and enzymes in urochordates^11^ and mammals^12^. Recent progress in genome research and sequence analyses has established that g-type lysozymes are as commonly distributed as chicken (c-) type lysozymes in the animal kingdom^13^,^14^,^15^.

Lysozymes or muramidases, play an important role in the innate immune system and catalyse the hydrolysis of the 1,4-beta-linkages between N-acetylmuramic acid (NAM) and N-acetyl-D-glucosamine (NAG) residues in bacterial cell wall peptidoglycans^16^. Ibrahim *et al.*^17^ also gave evidence for retained antibacterial activity for lysozymes catalytically inactivated by substitution of an active site residue. In addition, previous reports have shown antibacterial activity for proteolytic fragments of both lysozymes^18^.

The antibacterial significance of lysozymes is further reflected by the presence of lysozyme-specific protein inhibitors in a wide variety of bacteria. The bacterial inhibitor of vertebrate lysozyme (Ivy) shows efficient inhibition of c-type lysozymes, and to a certain degree also of g-type and phage lysozymes^19^, but not capable of inhibiting the enzymatic activity of fish g-type lysozymes^20^. However, a highly specific bacterial inhibitor of g-type (PliG) lysozymes was recently identified^21^.

Lysozymes are commonly found in combinations of two types in a species. Invertebrates typically have the i-type^22^ together with c- or g-type lysozymes. A surprising exception was the finding that protochordates carry multiple g-type genes, but no c- or i-types of lysozyme^11^. Most vertebrates studied to date possess both c-type and g-type genes^13^,^14^,^15^. The majority of reports on fish lysozymes show that these two types of lysozymes possess different roles and functions in antibacterial defense. Fish c-type genes are frequently expressed in a constitutively housekeeping fashion while g-type gene expression is stimulated in an infection-response manner by bacteria or bacterial components, although the Atlantic salmon shows the opposite lysozyme expression profile^23^.

A recent genome survey by Irwin^14^ identified 234 single or duplicated lysozyme g-type sequences from 118 vertebrate species representing all vertebrate classes except cartilaginous fish. Phylogenetic analyses indicated that most of these gene duplicates are recent or lineage specific events. Genome sequences of two bony fish (gar and tilapia) revealed no g-type lysozyme genes, while single or multiple genes for the g-type were found in the genomes of other teleosts. The cod had the largest number of g-type lysozyme genes, with as many as 11 potential genes with seven of these genes clustered on two scaffolds and the remaining four on separate contigs, a distribution making the exact numbering of functional genes uncertain. However, only two full-length cod g-type lysozyme genes, named LygF1b and LygF1d and residing on different scaffolds, could be deduced from the genome, and they apparently arose from a relatively recent cod lineage-specific gene duplication event^14^. LygF1b is identical to the previously reported cod lysozyme gene and its partly characterised antibacterial recombinant product^24^. This gene contains alternative transcription start sites that give rise to two gene products with or without an exon-1 encoded signal peptide for secretion. The sequence of the second g-type lysozyme, LygF1d, possesses substitutions at the active site residue^14^. The active site glutamate residue is crucial for catalytic activity and structural stability of g-type lysozymes. Substituting this residue with alanine, glutamine and aspartate abolish (Ala and Gln) or drastically reduce (Asp) the lysozyme enzyme activity^25^. Thus, the Glu ->Ala substitution in LygF1d should prevent enzymatic activity. Furthermore, two aspartate residues nearby the active site in the LygF1b cod g-type lysozyme seem central to ensure that a water molecule is in a proper location to perform the nucleophilic attack during catalysis. Substitution of any of these two aspartates reduce the enzyme activity 10–300 fold^26^.

No report so far has indicated the absence of a c-type lysozyme in cod or other gadoids. The present study shows that lysozyme activity from the cod belongs to the g-type only, and that sequence homology searches revealed a lack of c-type lysozyme genes in the cod genome. Analyses of four identified g-type lysozymes LygF1a-d demonstrated that multiple tissues express all four genes. Expression of only the two enzymatic low-active or inactive LygF1c and d are stimulated by an intracellular bacterium that commonly infects cod. This up-regulation is further enhanced by interferon gamma (IFNγ). Expression of none of the genes is affected by the bacterial component lipopolysaccharide (LPS) suggesting an intracellular function for LygF1a, c and d.

## Results

A goose-type lysozyme in gadoids was for the first time reported in 2009^24^ and recently identified by Irwin as one (annotated LygF1b) out of 11 potential g-type lysozymes in cod^14^. There is, however, no work on gadoid c-type lysozymes published to this day. The present work focuses on identification of any cod lysozyme with apparent functional role based on enzyme/muramidase activity, protein structure, gene appearance, gene expression and expression regulation.

### Identification of lysozymes genes in cod

Protein sequences of c-type lysozymes from chicken, human and eight fish species were used in TBLASTN searches for similar putative translation product sequences in the cod genome. Unexpectedly, these analyses revealed no indication for the presence of a c-type lysozyme gene in cod.

**Figure 1:**
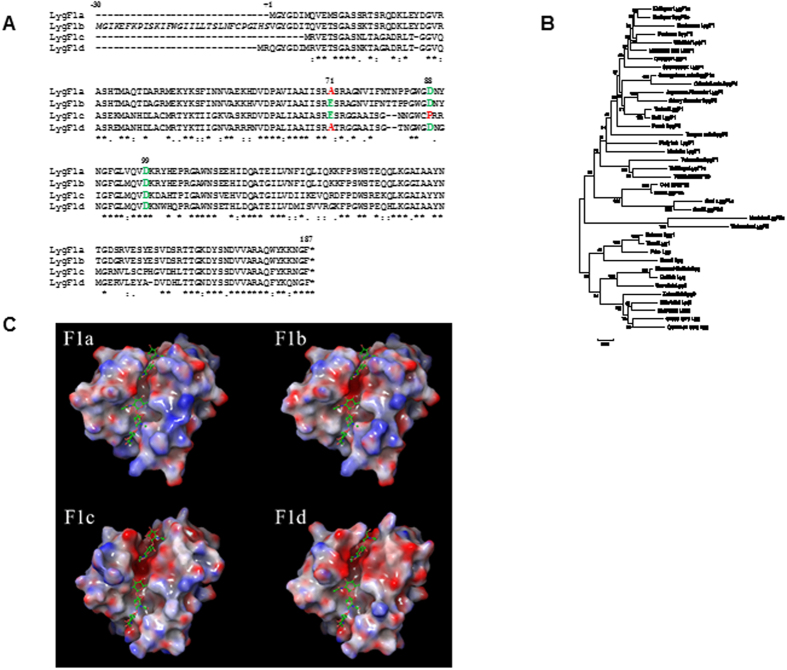
Atlantic cod g-type lysozyme proteins and electrostatic potential. (**A**) Alignment of cod g-type lysozyme protein sequences. Amino acid sequence alignment was generated with Clustal Omega, with the symbols below the alignment complete conservation (*), sites with strongly conserved properties (:), and sites with weakly conserved properties (.). The sequences are numbered from the N-terminus of the mature LygF1b proteins, with the signal peptide in italic and numbered backwards. The catalytic residues equivalent to E73 (E71 in the cod LygF1a/LygF1b sequences) and D86 and D97 (D88 and D99 in the cod LygF1c/LygF1d sequences) in the g-type sequence are indicated, with residues compatible with enzymatic function in green and those that should prevent enzymatic activity shown in red. **(B)** Phylogenetic relationships of g-type lysozyme from cod and other fishes. A bootstrapped (1000 replications) neighbour-joining tree based on maximum composite likelihood distances was generated using MEGA6.2^54^. Numbers at the nodes represent the number of bootstrap replicates (out of 1000) that supported each node. Branch lengths are proportional to amount of inferred change, with scale bar (changes per base) shown at the bottom. Trees were rooted based on previous phylogenetic analyses of g-type lysozyme sequences^14^. (**C**) 3-D models of LygF1a, LygF1c, and LygF1d were constructed based on the previously identified structure of LygF1b^26^ and the electrostatic surface potential is indicated in red (negative) and blue (positive). NAG molecules are presented as sticks docking into the LygF1 models in correspondence with the observations in native LygF1b.

In contrast, BLAST searches using the previously identified g-type lysozyme from cod^24^ and salmon^27^ as query sequences identified three complete and one near-complete genes encoding the g-type lysozymes previously annotated as LygF1a-d^14^ (Fig. 1A) contained in gene scaffolds 3789 and 1808. Searches of EST databases identified multiple expressed sequences from each of the three cod genes LygF1b, c and d. Two ESTs that show 5′ or 3′ sequence overlap with the partial LygF1a gene sequence allowed identification of a complete gene and the prediction of its complete encoded sequence. The extended LygF1a cDNA sequence indicated that the contig containing the previously annotated LygF1i sequence^14^ must be located near gene scaffold 3789, which encodes the remainder of LygF1a, in the cod genome. We also revealed a fifth potential full length cod g-type lysozyme gene, previously designated LygF1j encoded by contig 587134^14^, but this gene was disregarded in the following work since no matching EST was found and we failed to identify any evidence for expression of this sequence.

### Atlantic cod goose-type lysozyme proteins

The LygF1b gene has two alternative transcription start sites, one of which results in a product containing a signal peptide that would allow secretion^24^. No signal peptide sequences were identified for LygF1a, c and d. The multiple protein sequence alignment in Fig. 1A displays that the active site glutamate residue E71 in the previously reported enzymatic active LygF1b is replaced by alanine A71 in LygF1a and LygF1d. Except for the proline P88 substitution in LygF1c, aspartate residues D88 and D99 involved in catalysis^26^ are conserved among the lysozymes. The predicted protein sequences of the cod g-type lysozymes suggest that only LygF1b and possibly LygF1c (although containing the aspartate → proline substitution; D88P) have enzymatic ability since LygF1a and d have mutated catalytic sites. Of worthwhile notice was one ESTs (EY974857.1) that matches LygF1d encodes a codon for glutamate at the position corresponding to E71, thus deviating from the A71 in the LygF1d gene, suggesting that this gene is polymorphic and that one allele may encode an active enzyme.

The LygF1a sequence is most similar to LygF1b, sharing 96 and 95% identity at the DNA and protein levels, respectively. LygF1c is closest to LygF1d in sequence (87 and 73% identical in DNA and protein sequence), with an average of 60% identity and 71% similarity seen between the two pairs of protein. All of the cod g-type lysozyme genes are more closely related to each other than to any other characterised fish g-type lysozyme sequence (Fig. 1B).

### Cod lysozyme structures

Previous structure studies of cod LygF1b revealed the presence of NAG in the substrate binding sites B-D and E-G^26^ at both sides of the catalytic glutamate E71, and that the two aspartate residues (D88 and D99 in Fig. 1A) are central for binding and positioning of a water molecule serving as a nucleophile and thus promoting muramidase activity^26^. To avoid speculations about potential enzymatic activity of LygF1a and d, carrying an alanine instead of a glutamate in the active site, we investigated the cod lysozyme 3-D structures based on models built from the crystal structure of LygF1b^26^. This investigation revealed only minor differences between the over-all structures of the four g-type lysozymes. As presented in Fig. 1C, the E-G binding cleft of LygF1c and d appears slightly wider than that of LygF1a and b and the charge of the surrounding surfaces are more negative in the LygF1c and d pair. Docking of NAG molecules, as observed in the native cod structure (PDB 3GXR), into the cod lysozyme binding clefts demonstrates sufficient space for ligand binding (ball and stick model in Fig. 1C). However, the static model provides no strong indication of any other residue nearby that can take over the nucleophilic role of E71 in LygF1a and d or that the expected negative effect of proline substitution of D88 in LygF1c is relieved by other residues. LygF1c displays additionally an even more open B-D binding site than the three others and carries several amino acid substitutions in the substrate binding sites compared to LygF1a, b and d in addition to the D88P substitution. Thus, it is possible that LygF1c has a substrate preference that may be different from LygF1a, b and d.

### Lysozyme activity and gene expression in cod tissues

**Figure 2:**
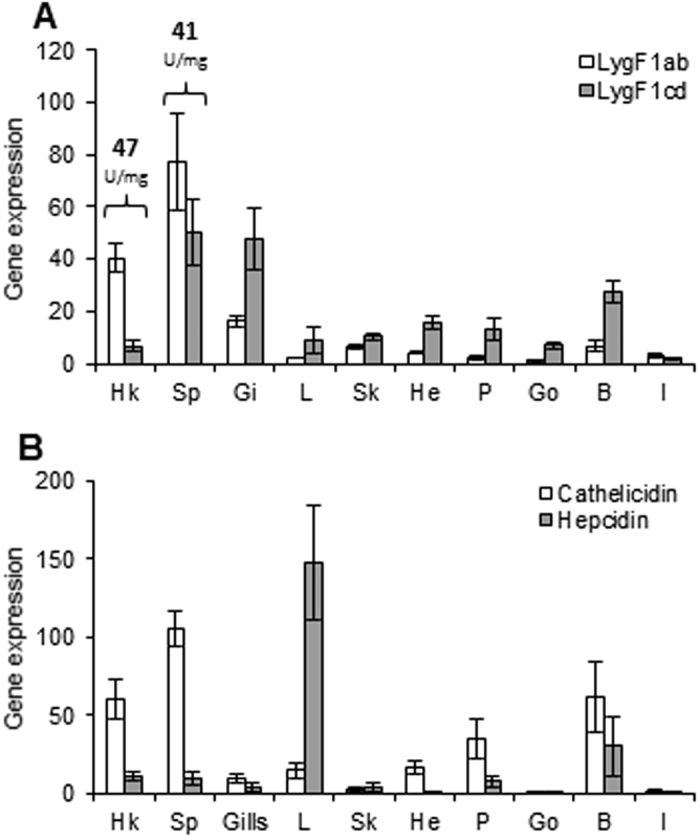
Constitutive expression of antibacterial genes in cod organs. Atlantic cod (100 g) head kidney (Hk), spleen (Sp), gills (Gi), liver (L), skin (Sk), heart (He), peritoneum (P), gonads (Go), blood (B) and intestine (I) were sampled and subjected to real time PCR analysis of gene expression for (**A**) g-type lysozymes LygF1ab and LygF1cd, and for (**B**) cathelicidin and hepcidin. Expression was normalised to the geometric mean of the reference genes (elongation factor eF1a, ubiquitin and 18S RNA) and calibrated to the organ (gonads) with lowest level of expression of antibacterial genes. Results are shown as relative quantification values obtained from four fish with mean quantity and calculated SEM. Total lysozyme enzyme activity measured is presented as Units mg^−1^ of total protein in spleen and head kidney (**A)** and is of the g-type exclusively. No lysozyme activity was detected in the other organs.

Lysozyme activities in extracts from spleen and head kidneys were determined in the absence and presence of the two specific bacterial lysozyme inhibitors Ivy and PliG^21^,^28^. All activity was suppressed in the presence of the g-type inhibitor PliG, while the Ivy c-type inhibitor had no effect on the lysozyme activity contained in these extracts (not shown). This shows that all measured lysozyme activity in these two organs arise from g-type lysozyme and at a level of 40–50 Units/mg total protein (Fig. 2A). No lysozyme activity was detected in the other organs examined.

PCR amplification of cDNA and subsequent sequencing of the four LygF1a-d lysozyme genes confirms their sequences as deposited in the cod genome database with one exception. Due to the discovery of an existing EST (EY974857.1) homologous to LygF1d, but carrying the active site glutamate residue, amplification products of LygF1d from four cod individuals were sequenced and one of the four confirmed the presence of this active site variant of LygF1d. We have no data on how frequent this presumably enzymatic active LygF1d variant occurs in nature.

The gene sequence of LygF1a differs from LygF1b in only 23 out of 564 nucleotides in positions scattered throughout the gene. This nearly full sequence identity represents a significant obstacle in analyses to discriminative gene expression of these two particular genes, although the longer 5′ gene sequence of the secreted form of LygF1b (including the encoded signal peptide) permits design of primers that selectively amplify its transcript. Initial work showed that PCR analyses of LygF1a gave significant footprints of LygF1b (secreted and non-secreted forms) as revealed by direct sequencing the respective amplified products. Even in the case of discriminating LygF1c from d, real time PCR co-amplification was observed although the DNA sequence identity between LygF1c and d is lower than between LygF1a and b. Thus, the following expression analyses ran primarily with primers that direct the co-amplification of LygF1a + b and of LygF1c + d. Consequently, we have also not tried to distinguish the two LygF1d variants regarding active site residue E71 or A71.

Real time PCR analyses revealed that both pair of genes LygF1a + b and LygF1c + d are expressed in all cod tissues albeit at different levels. The hematopoietic organs, head kidney and spleen, showed the highest expression of LygF1a + b where also lysozyme activity was detected (Fig. 2A). Although lysozyme activity was detected only in head kidneys and spleen, g-type lysozyme gene expression dominated by LygF1c and d is considerable in organs such as gills and blood.

Expression of two other antibacterial genes, cathelicidin and hepcidin, was included in this tissue distribution study for comparison to lysozyme (Fig. 2B). Cathelicidin is expressed at a significantly higher level (5–10-fold) than hepcidin in the hematopoietic organs.

### Gene expression after LPS treatment

**Figure 3:**
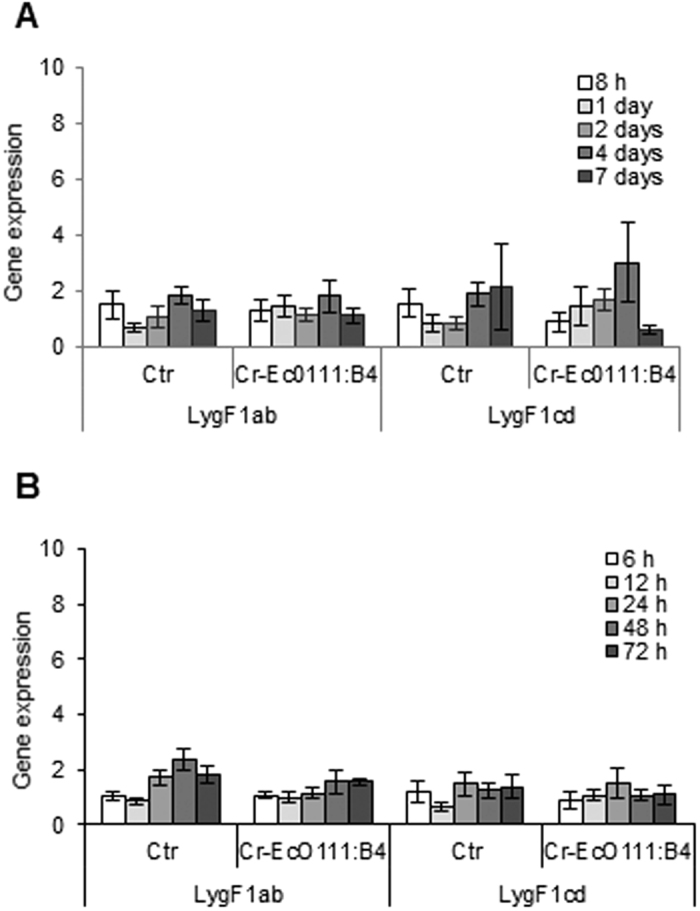
Gene expression of lysozymes in cod subjected to LPS. (**A)** Atlantic cod (approx. 50 g) were injected with 1 mg/kg crude (Cr) *E. coli* (Ec) LPS (0111:B4) or control (0.9% NaCl). Head kidney were sampled after 8 h, 1, 2, 4 and 7 days and subjected to real time PCR analysis of the LygF1 lysozymes. Expression of target genes were normalised to 18S RNA expression and calibrated to non-injected fish. Results are shown as relative quantification values obtained from six fish with mean quantity and calculated SEM. (**B)** Monocytes/macrophages were stimulated with Cr-Ec0111:B4 or left untreated (Ctr) and analysed for gene expression after 6, 12, 24, 48 and 72 h. Expression of target genes were normalised to eF1a expression and calibrated to untreated control at 6 h. Results are shown as relative quantification values obtained from four fish with mean quantity and calculated SEM.

We have recently shown that *in vitro* and *in vivo* expression of Atlantic cod hepcidin and cathelicidin is highly induced by LPS^29^, a commonly used bacterial Pathogen Associated Molecular Pattern (PAMP), and that *in vitro* expression of Atlantic salmon c-type lysozyme unlike the g-type is enhanced in the presence of LPS^23^. Here, gene expression of the cod g-type lysozymes was studied after injection of crude *E. coli* LPS into live fish or after co-incubating macrophages with LPS. No LPS-inducible cod g-type lysozyme expression was detected in head kidneys tissue samples or in the macrophages (Fig. 3). Similarly, crude LPS isolated from *F. noatunensis* had no effect on the *in vivo* expression of lysozyme in cod (not shown).

### Gene expression after *F. noatunensis* infection *in vivo*

**Figure 4:**
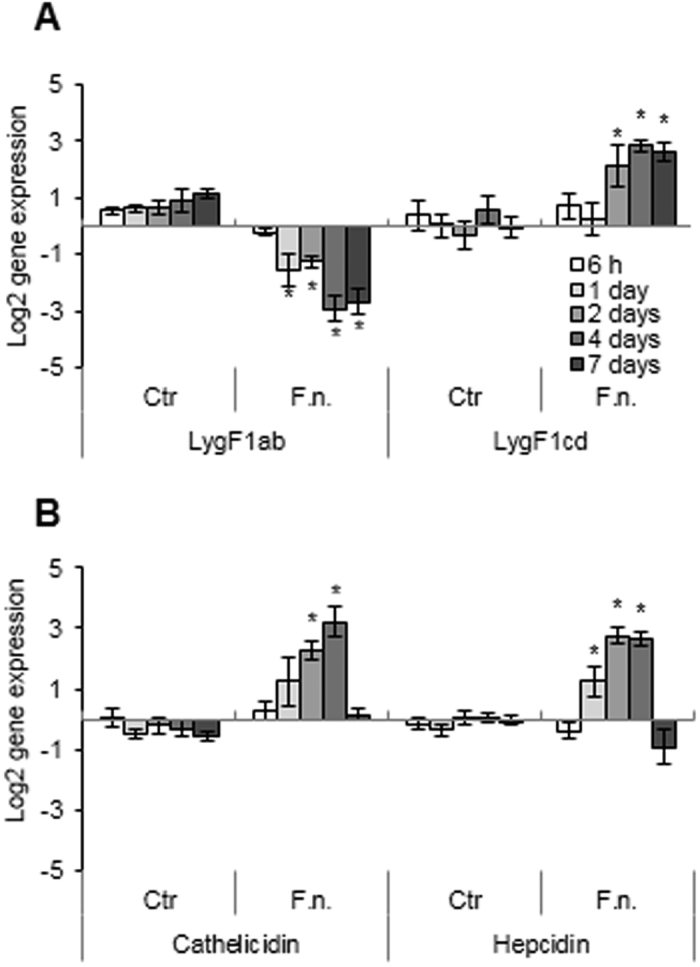
Expression of antibacterial genes in Atlantic cod head kidney after injection with *F. noatunensis*. Atlantic cod (approx. 25 g) were injected with 5 × 10^7^ *F. noatunensis* (F.n.) or control (0.9% NaCl). Head kidney were sampled after 6 h, 1, 2, 4 and 7 days and subjected to real time PCR analysis of (**A**) LygF1ab and LygF1cd lysozymes, and of (**B**) cathelicidin and hepcidin. Expression of target genes were normalised to 18S RNA expression and calibrated to non-injected fish. Results are shown as relative quantification values obtained from six fish with mean quantity and calculated SEM. The asterisks above columns indicate significant differences (p < 0.05) compared to the control at the same sampling point.

Atlantic cod were injected i.p. with the facultative intracellular bacteria *F. noatunensis* to study the responding effect on the transcription of antibacterial genes in the host. Somewhat surprisingly, gene expression of LygF1a + b was significantly down-regulated 1–7 days after injection of bacteria. LygF1c + d expression, on the other hand, was significantly up-regulated at 2, 4 and 7 days after injection (Fig. 4A). In the amplified LygF1c + d pair, both genes have higher expression levels in infected cod although LygF1d appeared to have the highest increase in expression. Expression of both antibacterial genes cathelicidin and hepcidin was significantly up-regulated after injection of bacteria, but returned to lower expression levels earlier than LygF1c + d (Fig. 4B).

### Effect of *F. noatunensis* infection and IFNγ on lysozyme gene expression *in vitro*

**Figure 5:**
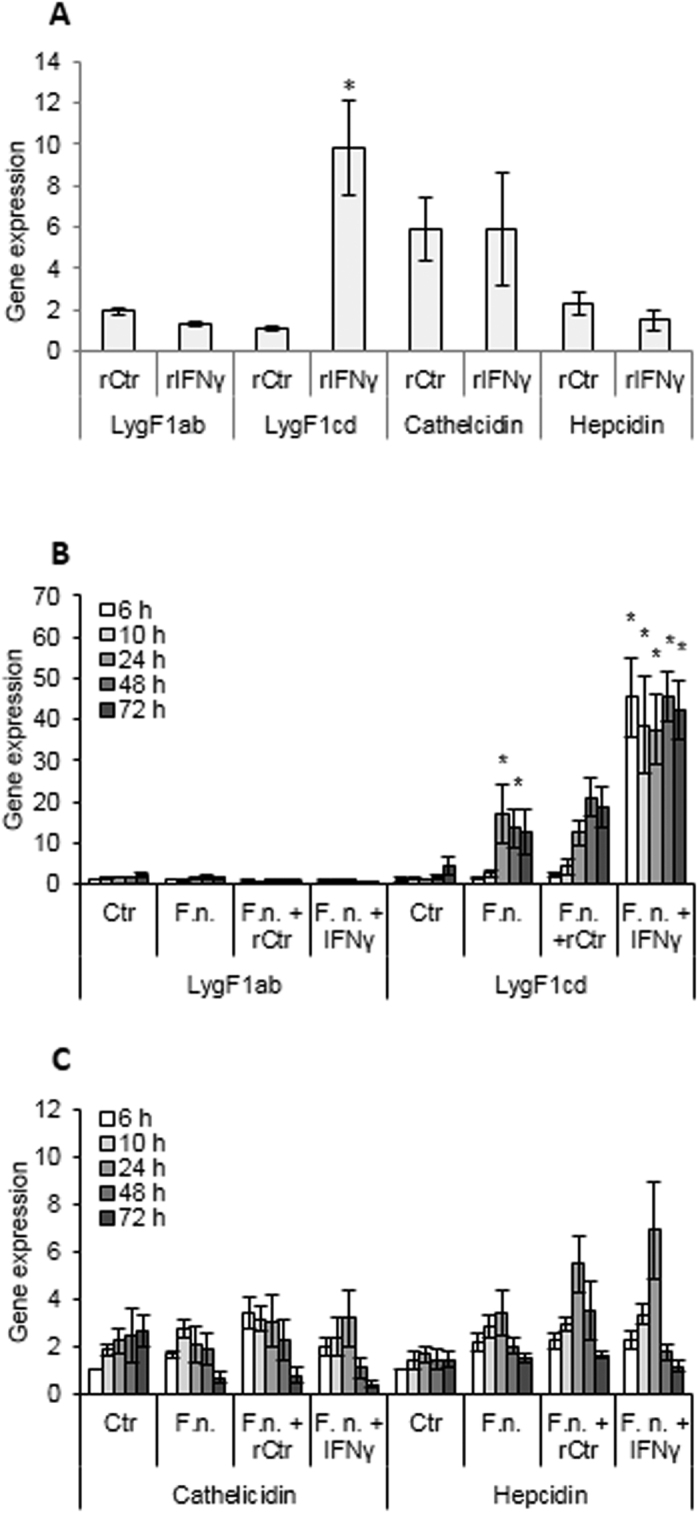
Antibacterial gene expression in monocytes/macrophages stimulated with IFNγ and infected with *F. noatunensis*. **(A)** Monocytes/macrophages were treated with recombinant IFN**γ** or recombinant control (rCtr; back-ground proteins isolated from *E. coli* containing an empty vector) and analysed for gene expression of g-type lysozymes (LygF1a + b and LygF1c + d) and antibacterial genes (cathelicidin and hepcidin). (**B,C**) Monocytes/macrophages were either infected with *F. noatunensis* (F.n.), pre-treated by recombinant rCtr or IFNγ prior to infection or left un-treated (Ctr) and subjected to gene expression of analyses (**B**) g-type lysozymes (LygF1a + b and LygF1c + d) and (**C)** and antibacterial genes (cathelicidin and hepcidin). Expression of target genes were normalised to eF1a expression and calibrated to the Ctr cells at 6 h. Results are shown as relative quantification values obtained from four fish with mean quantity and calculated SEM. The asterisks above columns indicate significant differences (p < 0.05) compared to the *F. noatunensis* infected cells at the same sampling point.

An infection study with *F. noatunensis* was also performed in cod macrophages and the decreased LygF1a + b expression (relative to uninfected control cells) seen *in vivo* following infection was less evident *in vitro* (Fig. 5). However, the bacteria-induced increase in LygF1c and d expression observed *in vivo* was reproduced in the *in vitro* cultures. Expression levels of all cod g-type lysozyme genes progressed in control cell cultures during the experimental period indicating an augmented amount of lysozyme as the macrophages mature.

The type II interferon IFNγ has a profound signalling and modulatory function on immune-related networks of genes and associated pathways^30^. Our studies showed that IFNγ mediated a stimulation of LygF1c + d in cod macrophages, while LygF1a and b expression remained at the control levels (Fig. 5). Evidently, IFNγ also enhanced the already stimulated expression of the LygF1c and d genes in cod macrophages infected with *F. noatunensis,* whereas LygF1a and b expression did not change in the IFNγ-treated infected cells. Hepcidin and cathelicidin expression was apparently unaffected by IFNγ and the slightly increased expression of hepcidin observed in cells infected by *F. noatunensis* was not statistically significant.

## Discussion

With a few exceptions, vertebrate genomes typically harbour both c-type and g-type lysozyme genes^13^,^14^. No g-type gene was found in tilapia, gar, and elephant shark genomes, while c-type lysozyme genes were not found in the avian zebra finch or the jawless lamprey fish^13^,^14^. However, these genes may exist in gaps in the available genome sequences of these species. Lysozyme activity has been described in a wide variety of vertebrates, nevertheless, the function of c-type lysozymes have been better characterised than of g-type lysozymes^15^,^31^. Although g-type lysozyme genes exist in diverse vertebrates, characterisation of g-type activity is dominantly from birds and fish. In contrast, c-type lysozyme activity is extensively studied in a wide variety of vertebrates^15^,^31^. Deficiency of c-type lysozyme has previously been reported in a few vertebrate species (e.g., birds, cow and rabbit^32^,^33^,^34^,^35^,^36^). For the mammalian species, these deficiencies were tissue-specific, with lysozyme being absent (or very low) in blood, tears, and/or milk, but with c-type lysozyme activity detected in other tissues^32^,^33^,^34^,^37^. Studies in birds have been more limited, with only egg-white examined in many species. Both c-type and g-type lysozymes are found in eggs, but in most species, only one but not the other^35^,^38^,^39^. Genome searches for avian lysozyme genes revealed the presence of g-type lysozyme genes in all 6 species examined^14^, while c-type lysozymes were found in only 3 of 4 species examined^13^, raising the possibility that the some birds, such as the zebra finch, may be missing a c-type lysozyme gene. Further searches of the genome and transcriptome of these birds, as well as examinations for enzymatic activity are needed to confirm the loss of the c-type lysozyme gene.

Here we conducted a more detailed examination of the genome, transcripts, and enzymes, and demonstrate for the first time a species, the cod, which truly lacks a c-type lysozyme gene. Despite previously demonstrated peculiarities in the cod immune system^4^, the present finding of a missing c-type lysozyme gene was quite unexpected. Cod differs from other teleost species in having highly amplified the g-type lysozyme gene, while the c-type lysozyme gene is absent in the cod genome. Intriguingly, a similar situation occurred in ruminant artiodactyl mammals, where the *LygA1* gene (a g-type lysozyme gene) was pseudogenized in parallel with the amplification of the c-type lysozyme gene^14^. These parallels suggest that amplification of one type of lysozyme gene may allow generation of redundant copies that can be neo-functionalized to replace the function of the second type of lysozyme gene, and allow the pseudogenization and/or gene loss of the second type of gene.

In contrast to LygF1b, the other intact g-type lysozyme genes in the cod carry either a substitution of the glutamate E71 (E71A in LygF1a and d) nucleophile positioned in the substrate binding cleft, or of the aspartate 88 (D88P in LygF1c) involved in water binding and catalysis that are conserved in enzymatically active g-type lysozymes^25^,^26^. Our structure models of these g-type lysozymes fail to identify any other nearby residues that are judged to have nucleophilic capacity to replace the role of E71 in catalysis. In other words, the primary and tertiary structures of LygF1a and d are not consistent with lysozyme enzyme activity according to the generally accepted catalytic mechanism and particularly according to the verified crucial role of E71 for catalysis by g-type lysozymes^25^. Furthermore, site-specific substitution of D88 in LygF1b with an alanine was earlier demonstrated to result in a 10-fold reduction of enzyme activity^26^, and the D88P substitution in LygF1c is expected to have at least similar negative impact on the enzyme activity.

The finding of a LygF1d variant carrying E71, and thus a presumably active enzyme, instead of A71 that removes activity is highly interesting. We have not conducted detailed studies on the relative expression between the two variants in the examined tissues. However, we are tempted to suggest that the LygF1d variant carrying E71 represents a minor allele based on the tissues distribution of lysozyme activity. From an evolutionary perspective, the LygF1d gene might represent an evolutionary intermediate that is undergoing the loss of muramidase activity, through the E71A substitution along with other possible amino acid substitutions, which is tolerated in cod due to the duplications of the g-type lysozyme genes and retention of a functional LygF1b gene that encodes muramidase activity.

Head kidney of the cod contains high levels of lysozyme enzymatic activity and our expression analyses shows that the LygF1b (or LygF1a + b) transcript level is almost 10 fold higher than LygF1c + d. Similar high levels of constitutive LygF1a + b and LygF1c + d gene expression were detected in the spleen. Other organs, e.g., gills and blood, contained LygF1c + d transcript levels equal to or slightly lower than the spleen, and with low levels for LygF1a + b. Detectable lysozyme enzymatic activity was only present in the head kidney and the spleen, organs where LygF1b (or LygF1a + b) expression dominates. These analyses are consistent with our conclusion that LygF1a, c and d are enzymatically inactive or low-active lysozymes, as suggested earlier for LygF1d^14^. We cannot exclude the possibility that there are additional functional cod g-type lysozymes among the remaining potential genes^14^, although BLAST searches of EST databases revealed no evidence for expression of any of these genes.

Unlike cod antibacterial hepcidin and cathelicidin^29^ and g- or c-type lysozymes of other fish species^23^, none of the cod lysozymes were transcriptional modulated by *in vitro* or *in vivo* administration of the bacterial cell wall LPS component. This does not necessarily mean that LPS has no immunomodulating effect on these lysozyme genes, e.g. these genes may require the mediation of LPS signals through intracellular routes different from those used by hepcidin and cathelicidin. If so, this is consistent with the idea that the studied g-type lysozymes have an intracellular role since they are clearly down or up-regulated by an intracellular infection.

Judged by previously reported real time PCR and “semi-quantitative PCR” experiments, formalin-inactivated and extracellular Gram-negative bacteria result in a slight increase in LygF1b transcription^24^,^40^. In the present study, infecting cod with facultative intracellular *F. noatunensis* provoked gene expression responses by both LygF1a + b and LygF1c + d, although they were in opposite directions. Recombinant IFNγ produced a similar *in vitro* stimulatory effect on LygF1c + d gene expression as seen by bacterial infection, with this cytokine giving a significant additive effect on LygF1c + d expression in infected cells. Thus, the LygF1c and d gene “pair” evidently responds as expected for genes of importance in an antibacterial system or pathway. Hepcidin and cathelicidin was, however, not affected by IFNγ or by infection.

IFNγ is acknowledged as a key activator of cell mediated immunity and especially important in defence against intracellular pathogens^41^. Another bactericidal mechanism induced by IFNγ is the production of different antimicrobial peptides (AMPs)^42^ and lysozyme^43^. While most AMPs are either secreted or delivered to phagosomes, AMPs are also found localised in cytosol^44^,^45^. *F. noatunensis* enters cod macrophages in membrane-enclosed vacuoles and most likely escapes to the cytosol for replication^46^ similar to *F. tularensis*. The observed gene expression kinetics for LygF1c + d thus could be consistent with a role for this lysozyme in inhibiting the intracellular growth of *F. noatunensis*. There are no previous reports on a differential influence of IFNγ on g-type lysozymes, but the challenge responses observed here support our conclusion that cod LygF1c and d possess qualities preferred, perhaps more than of the enzymatic active LygF1b, for the defence against intracellular bacteria. The mechanisms limiting cytosolic growth of bacteria are not well understood, but intracellular localised lysozyme in concert with IFNγ might serve an important role in controlling intracellular growth of *Francisella*, a hypothesis that deserves further investigation.

Duplication of the g-type lysozyme genes and retention of non-secreted isozymes that have lost enzymatic activity was proposed to indicate that these lysozymes had roles other than being an antibacterial enzyme through its associated muramidase activity^14^. Evidence for a potential function for these lysozymes come from the observations of significant antibacterial activity in enzymatically-inactivated lysozymes^17^ and for proteolytic fragments, or synthetic peptides, derived from lysozyme proteins^18^. In these cases, the membrane-penetrating capacity of the lysozyme or lysozyme-derived fragment mediates the antibacterial action. Lysozymes also promote aggregation of bacteria by binding to the bacterial surfaces^47^. Aggregation *per se* may have limited antibacterial effects, but it will ease the recognition and engulfment of lysozyme-bound bacteria.

Bacteria are one of the targets of selective autophagy, and this process is known as xenophagy (degradation of microorganisms). In this context, autophagy acts as an innate immune mechanism against bacterial infection. Autophagy paradoxically also represents a survival/escape route for some pathogens^48^. Extensive work has been done to determine the induction and targeting mechanisms of antibacterial autophagy believed to involve activation of multiple host factors and pathways. Selective autophagy implies cargo selection mediated by various receptors and adaptor proteins. For instance, the autophagy receptor p62 links various ubiquitinated cargo targets including bacteria to autophagosomes^49^, and NBR1 is involved as adaptor for the bacterial pathogen *F. tularensis*^50^. The existence of multiple intracellular cod g-type lysozymes with no or very low enzyme activities, and the demonstrated modulation of lysozyme gene expression by bacterial infection and IFNγ, allows speculations about a possible role for lysozymes in the process of selective autophagy of bacteria.

The four cod g-type lysozymes investigated in this study are LygF1a-d of which only LygF1b was previously identified as an active enzyme^24^, while the recent characterisation of LygF1d was restricted to comparative sequence analyses^14^. Our results show that the LygF1a, c and d genes are constitutively expressed at a significant level in all organs examined, and the level of expression was even higher than that of LygF1b in several of the organs. Based on the antibacterial nature of lysozymes, or at least their indisputable part in the innate defense system, the wide tissue distribution of the non-enzymatic or low-active LygF1a, c and d strongly suggests that these g-type lysozymes have functional roles in cod innate immunity. As shown by infection studies, regulation of gene expression differed from LygF1a + b to LygF1c + d, and modelled 3-D structures implied some differences in substrate binding capacities, in particular for LygF1c. Taken together, this indicates not only that the multiple g-type lysozymes play functional roles, but also that their roles differ.

The present work shows that c-type lysozyme is not an essential enzyme, although duplication of the g-type lysozyme may have allowed the evolution of a g-type lysozyme that could functionally replace the missing c-type lysozyme. Which of the four g-type lysozymes, the enzymatically active or those inactivated by active site substitutions, is functionally compensating for the loss of the c-type lysozyme still needs to be identified. Studies in species related to the cod should help, by determining the times of the loss of the c-type lysozyme gene, duplication of the g-type lysozyme genes, and the mutational changes that prevent enzymatic activity.

## Materials and Methods

### Search for c- and g-type lysozyme genes and transcripts

Searches for c- and g-type lysozymes sequences using the basic local alignment search tool (TBLASTN^51^) were performed on the Ensembl Atlantic cod genome database (http://www.ensembl.org/Gadus_morhua) and in the European Nucleotide Archive (ENA) for expressed sequence tags using NCBI BLAST+ (http://www.ebi.ac.uk/Tools/sss/ncbiblast/nucleotide.html). In the searches for cod c-type genes or transcripts, a panel of c-type query sequences from the following sources were employed; fugu (P61944), starry flounder (BAL44624.1), spotted green pufferfish (AG06232.1), Senegalese sole (ABC49680.1), turbot (CE80211.1), rainbow trout (AF321519.1), zebrafish (NP631919.1), Atlantic salmon (ACM09320.1), human (NP000230.1) and chicken (P00698). Similar searches for g-type lysozymes were conducted using a previously characterised cod g-type lysozyme (EU377606^24^) and the Atlantic salmon g-type lysozyme (CAM35431^27^). A multiple sequence alignment of the proteins was constructed using Clustal Omega with default settings^52^ and subsequently edited using GeneDoc, version 2.7^53^.

### Phylogenetic analysis

Phylogenetic relationships among the cod sequences and g-type lysozymes from other fish were assessed using the neighbour-joining method using MEGA6.2^54^. Nucleotide sequences of g-type lysozyme from cod and other fish were aligned at the codon level (to maintain coding potential) using Muscle as implemented in MEGA6.2^54^. Bootstrapped neighbour-joining trees, 1000 replications, were generated using maximum composite likelihood distances. Trees were rooted based on previous phylogenetic analyses of g-type lysozyme sequences^14^.

### 3-D structure modelling

Homology models of LygF1a, c and d were generated by feeding the edited alignment and native structure of cod lysozyme (PDB 3GXK^26^) into the Maestro v9.4 option of the Schrödinger software (Schrödinger, LLC, NY, 2013).

### Cod tissue sampling

Atlantic cod (100 g; n = 4), healthy and without any signs of disease, were obtained from the Aquaculture Research Station (Tromsø, Norway). The use of live Atlantic cod was approved by the National Animal Research authority in Norway and all methods were in accordance with the approved guidelines. The fish were kept in circular 1500 L tanks in seawater (3.4%) at natural seawater temperature (3–9 °C) and fed *ad libitum* with commercial feed (Amber Neptun; Skretting) under a 24 hour light regime. Fish used for our studies were rapidly killed and blood was removed by bleeding the fish from the *Vena caudalis*. Following sampling, cod organs (head kidney, spleen, gills, intestine, liver, skin, heart and peritoneum) were split in two parts and submerged in RNAlater (Ambion) for subsequent gene expression studies or stored at −20 °C for later enzyme activity measurements. Blood for gene expression studies was sampled in 1x lysis buffer (Applied Biosystems).

### Lysozyme activity measurements

After thawing on ice, tissue samples were homogenized in 0.5 ml or 1 ml of 0.1 M sodium acetate buffer, pH 6, using a Polytronic PT 1200 handheld device. The extracts were then centrifuged at 20,000 × *g* for 20 min and the supernatants subsequently subjected to lysozyme activity measurements. Assays were run at 22 °C using 0.3 mg/ml lyophilised *Micrococcus luteus* as a substrate in sodium acetate buffer (0.1 M; pH 4.8) and subsequent recording of change in absorbance at 450 nm with time. One unit of activity was defined as the amount of enzyme that catalyses a decrease in absorbance of 0.001 min^−1^. The effect of bacterial lysozyme inhibitors on tissue lysozyme activity was performed in the same assay using 1 μg of the bacterial inhibitors of c-type lysozyme (Ivy) or g-type lysozyme (PliG)^21^,^28^ representing an excessive concentration compared to the contained lysozyme activities. The inhibitors were included in the substrate mixture before adding enzyme-containing extract for recording of activity.

### Isolation of monocyte/macrophages from Atlantic cod

Head kidney derived macrophages were isolated from cod based on previously described protocols. In short, head kidneys were aseptically removed and transferred to L-15++ (L-15 (Gibco, Invitrogen or PAA Laboratories) supplemented with 25 mM HEPES, 2 mM l-glutamine, 13.7 mM NaCl, 1.8 mM glucose, 4.2 mM NaHCO_3_, 20 U/ml penicillin, 20 μg/ml streptomycin and 10 U/ml heparin (LEO Pharma AS)). Head kidney were homogenised (GentleMACS^TM^ dissociator, Miltenyi Biotec) and minced through a 100 μm nylon Falcon cell strainer (BD Bioscience) and diluted in 90 ml L-15++. Cell suspensions were loaded on discontinuous 28%/45% Percol (GE Healthcare) gradients and separated by centrifugation at 400 × *g* for 40 min at 4 °C. The interphase containing purified macrophages was washed twice in 50 ml L-15+ (L-15++ without heparin) followed by centrifugation at 300 × *g* for 10 min at 4 °C. In the last washing step, cells were diluted in L-15+ and seeded at a density of 5–10 × 10^6 ^cells per well in 24-well culture plates (Nunc or BD Biosciences). Covered by Microplate sealing Tape (Nunc), the culture plates were incubated at 12 °C to the following day when cells were washed twice in L-15+ for further studies.

### Stimulation of cod monocytes/macrophages with LPS

Stimulation of primary cultures with LPS is described elsewhere^29^. In short, monocyte/ macrophage cultures (n = 4) were stimulated with 20 μg/ml crude *E. coli* LPS (0111:B4; Sigma-Aldrich) for sequential time points (6, 12, 24, 48 and 72 h) and cells were harvested by adding 1x lysis buffer (Applied Biosystems). All samples were stored at −80 °C for further gene expression analysis as described below.

### Injection of Atlantic cod with LPS

Fish (approx. 50 g) kept at 10 °C were fed *ad libitum* using a commercial feed (Amber Neptun; Skretting). The *in vivo* injection study has been described previously^29^ and was approved by the National Animal Research authority in Norway (FOTS Id 3033). In short, cod were anaesthetised with Metacainum (50 mg/l, Norsk Medisinaldepot) and injected intraperitoneally (ip) with 100 μl of 0.9% NaCl (Ctr) and crude *E. coli* LPS (0111:B4; 2 mg/kg) diluted in 0.9% NaCl. A control group that was not injected was also sampled. Fish (n = 6 for each time point) were sampled after 8 h, 1, 2, 4 and 7 days in RNA-later (Ambion), kept at 4 °C overnight before storage at −80 °C.

### Injection of Atlantic cod with *F. noantunensis*

The *Francisella noatuensis* subsp. *noatuensis* NCIMB 14265^55^ isolate used for challenge was originally isolated from diseased Atlantic cod (*Gadus morhua*) in Norway, and were provided by Dr. Duncan Colquhoun at the National Veterinary Institute Oslo, Norway. The bacteria were cultivated at 21 °C for 7–10 days on CHAB agar^56^ and heart infusion broth (Merck) pH 6.8 ± 0.2, supplemented with cysteine 0.1% (Merck, Germany), haemoglobin (Oxoid, England), 2%, glucose 1%, agar 1.5% and 5% human blood concentrate. The bacterium was stored in glycerol cultures at −80 °C. Pure colonies were inoculated in Bacto heart infusion broth (Becton and Dickson, USA) pH 7, supplemented with cysteine 0.07%, FeCl_3_ 2 mM and glucose 1%, and incubated with agitation at 21 °C for 24–30 hours before used in the challenge study. CHAB plates were used for determination of colony forming units (cfu) of challenge dose. The fish were reported to be healthy without any history of diseases and all efforts were made to minimise suffering. The experiment was approved by the National Animal Research authority in Norway (FOTS Id 1147).

Fish (approx. 25 g) acclimated to 15 °C and starved 24 h were anaesthetised with Metacainum (50 mg/l, Norsk Medisinaldepot) prior to i.p. injection (100 μl) of either *F. noatunensis* (5 × 10^7 ^cfu per fish) or 0.9% NaCl (control). At fixed time intervals after injection, fish were rapidly killed and blood was removed by bleeding the fish from the *Vena caudalis*. Head kidney (n = 6 for each time point) were sampled at 0, 6 hours, 1, 2, 4 and 7 days post infection. No mortality was recorded in any of the tanks.

### Recombinant production of IFNγ and activation of macrophages

The open reading frame of the DNA sequence for cod IFNγ (accession number FJ356236.1) was inserted into the expression vector pUC57 containing an N-terminal 6-His tag (Genscript, NJ). To allow expression of the recombinant protein, the plasmid was transformed into *E. coli* cells and recombinant (r) IFNγ was purified using Ni-HiTrap column (Genscript, NJ). The purified protein was resolved by 4–20% SDS-PAGE and visualised by Coomassie Blue staining. Protein concentration was determined by comparing the protein band density with a standard protein. Western blot analysis was performed to confirm the identity of the rIFNγ using Anti-His antibody revealing a single band. A plasmid without insert was transformed into *E. coli* and the bacterial extract was purified similarly as rIFNγ to be used as control in the *in vitro* experiments (rCtr). The endotoxin level was determined to be < 0.25 Eu/μg LPS by the LAL method. In order to study the induction of gene expression by rIFNγ, isolated macrophages (n = 4) were treated for 24 h with 1000 ng/ml rIFNγ and rCtr (diluted similarly). Cells were harvested in 1x lysis buffer (Applied Biosystems) and RNA was isolated for gene expression analysis of LygF1a + b and LygF1c + d as described below.

### Infection of cod monocytes/macrophages with *F. noantunensis*

Monocyte/macrophage cultures (n = 4) were pre-treated for 24 h with IFNγ at a concentration of 1000 ng/ml or left un-treated (Ctr). Cells were infected with *F. noatunensis* (NCIMB 14265) diluted in L-15+ with 5% FBS Gold (PAA) at a multiplicity of infection (MOI) of 50–100. Uninfected cells were incubated with cell media/FBS Gold alone and treated otherwise similarly as infected cells. The cell culture plates were centrifuged (500 × *g,* 5 min) to enhance the initial contact with the cells. Two hours after infection, the cells were washed three times in L-15++ and pulse-treated for 1 h with 50 μg/ml gentamicin (Sigma-Aldrich) to kill extracellular bacteria. The cells were washed three times to remove gentamicin and fresh media supplemented with 5 μg/ml gentamicin (Sigma-Aldrich) were added. Cell cultures were further incubated before harvesting in 1x lysis buffer (Applied Biosystems) at sequential time points (6, 10, 24, 48 and 72 h). All samples were stored at −80 °C for further gene expression analysis as described below.

### Gene expression analysis

Cells were harvested in 1x Nucleic Acid Purification Lysis buffer as described above, while tissues were homogenised in 1x lysis buffer using MagNA Lyser Green Beads and the MagNa Lyser Instrument (Roche Diagnostics). Total RNA was isolated using an ABI Prism 6100 Nucleic Acid Prep Station (Applied Biosystems) with the recommended on-column DNAse treatment. Reverse transcription was performed using the High capacity RNA to cDNA master mix or High capacity cDNA reverse transcription kit (Applied Biosystems) with the addition of 2.5 mM poly dT primer (Promega). The reaction conditions were 25 °C (5–10 min), 42 °C (60–120 min) and 85 °C (5 min) and the cDNA was diluted 1:30 in nuclease free water (Ambion) for further use in quantitative real time PCR. The absence of genomic DNA in the RNA was verified by real time PCR analyses without prior cDNA synthesis on a selection of samples from each experiment. Real time PCR was performed in duplicates in 384 well plates using the 7900HT Fast real-time PCR system and Power SYBR green PCR master mix according to the manufacturers description (Applied Biosystems). Real time PCR primers are listed in Table 1 with references for previous publications or as GenBank accession numbers for primers designed for this study. Gene expression data were analysed with the SDS 2.3 software (Applied Biosystems) and exported to Microsoft Excel for further analysis. The efficiency of the PCR reactions was determined by linear regression analysis of 2-fold dilutions of cDNA (total RNA isolated from cod head kidney) and denoted with a correlation coefficient (r^2^). Quantification of relative gene expression levels were performed using the 2^−∆∆CT^ method^57^. For quantification of target genes expression among different organs, the geometric mean of the three reference genes elongation factor 1α (eF1a), ubiquitin and 18S RNA was used for normalisation, while eF1a and 18S RNA was used for normalisation of *in vitro* and *in vivo* expression experiments, respectively. After normalisation, the expression level was calibrated to non-stimulated/non-infected or uninfected controls. From relative quantification values obtained from cells or fish, the mean quantity ± SEM was calculated. Statistical analyses between groups were made with the Student *t*-test and *P* < 0.05 was considered significant.

### DNA Sequencing

Sequence analysis of the four lysozyme transcripts was performed on cDNA (1/50 dilution) from the different experiments in this study. Transcripts regions were amplified by PCR using a combination of specific primers and mutual primers. Primer sequences were as follows: LygF1a; Lyg1Fa-26F (5′-TCAAACCGATGCTCGGAGG-3′) and LygF1ab-560R (5′-CTAAAACCCGTTTTTTTTGTACC-3′), LygF1b; Lyg1Fb-197F (5′-CTCAAACCGATGCTGGGAGA-3′) and LygF1ab-560R (5′-CTAAAACCCGTTTTTTTTGTACC-3′), LygF1c; Lyg1Fc-132F (5′-TGGTGTACAGGCATCGGAAA-3′) and LygF1c-537R (5′-CTAAAACCCGTTTCGTTTGTAAA-3′), LygF1d; Lyg1Fd-133F (5′-GGTGTACAGGCATCGCGAGA-3′) and LygFd-576R (5′-CTAAAACCCGTTTTGTTTGTAAAA-3′).

PCR was carried out using Q5 high-fidelity 1x master mix (New England Biolabs) and 500 nM of each primer (Sigma-Aldrich) in a total volume of 25 μl. The PCR thermal conditions were initial denaturation at 96 °C for 30 s and 35 cycles at 96 °C for 5 s, 60 °C for 10 s, 72 °C for 20 s followed by a final extension at 72 °C for 2 min. Non-incorporated primers and nucleotides were digested with HT ExoSAP-IT (Affymetrix USB) according to the manufacturer recommendations.

Sequencing was performed on an ABI 3130xl genetic analyzer (Applied Biosystems) using the Big Dye Terminator v3.1 Cycle Sequencing Kit (Applied Biosystems) according to the manufacturers instructions. Sequencing primers were identical to the PCR-primers. The obtained sequences were aligned using the SeqMan program within the Lasergene software package (DNASTAR Inc).

## Additional Information

**How to cite this article**: Seppola, M. *et al.* Multiple specialised goose-type lysozymes potentially compensate for an exceptional lack of chicken-type lysozymes in Atlantic cod. *Sci. Rep.* **6**, 28318; doi: 10.1038/srep28318 (2016).

## Figures and Tables

**Table 1:** Real time PCR primers, PCR efficiency and correlation coefficient (r^2^).

Gene name	Sequence (5′-3′)	PCR efficiency/r^2^	Reference
LygF1b (secreted)	GAGTTCAAGCCAATCTCCAAGATATT	95.5%/1.000	This study
(EU377606)	GATGTCTCCGTACCCTACAGAATGA		
LygF1a + b	TTCGCGACAGGATAAACTGGA	96.2%/0.999	This study
(EU377606)	TTGTATTTTTCCATTCTCCCAGC		
LygF1c + d	GGCCAACCATGATTTGGCTT	94.7% /0.999	This study
(ENSGMOT00000015279)	AGCTGGGTCAACATTACGTCTGC		
Cathelicidin	CACAAGAGTTAGACTGCAGCCAAG	96.2%/1.000	58
(EU707291.1)	TGTAGCTCAGGGTGAAATTGCAAT		
Hepcidin	CCAGAGCTGCGGATCGA	99.4%/1.000	59
(EU334514.1)	AAGGCGAGCACGAGTGTCA		
Elongation factor 1α	ATGTGAGCGGTGTGGCAATC	96.4%/1.000	60
(DQ402371.1)	TCATCATCCTGAACCACCCTG		
Ribosomal RNA (18S)	GAGCCTAGAAATGGCTACCACATC	93.3%/0.998	60
(U11437.1)	CACGTGTCGTGAATGGGTAATT		
Ubiquitin	GCCGCAAAGATGCAGAT	91.2%/0.995	61
(EX735613)	CTGGGCTCGACCTCAAGAGT		
